# Microbial biofilms and the human skin microbiome

**DOI:** 10.1038/s41522-016-0004-z

**Published:** 2016-11-23

**Authors:** Michael Brandwein, Doron Steinberg, Shiri Meshner

**Affiliations:** 1 Faculty of Dental Medicine, The Hebrew University of Jerusalem, Hadassah Ein Kerem, Jerusalem, Israel; 2 Dead Sea and Arava Science Center, The Dead Sea Microbiology lab, Ein Gedi, Israel

## Abstract

The human skin microbiome plays an important role in both health and disease. Microbial biofilms are a well-characterized mode of surface-associated growth, which present community-like behaviors. Additionally, biofilms are a critical element in certain skin diseases. We review how the perception of the resident skin microbiota has evolved from the early linkages of certain microbes to disease states, to a more comprehensive and intricate understanding brought on by biofilm and microbiome revelations. Rapidly expanding arsenals of experimental methods are opening new horizons in the study of human–microbe and microbe–microbe interactions. Microbial community profiling has largely remained a separate discipline from that of biofilm research, yet the introduction of metatranscriptomics, metabolomics, and the ability to distinguish between dormant and active members of a community have all paved the road toward a convergent cognizance of the encounter between these two microbial disciplines.

## Introduction

Bacteria adapt to life on surfaces through the induction of a number of metabolic changes, including the production of an extracellular substance to hold bacterial communities together and the regulation of certain genes through quorum sensing. In this state, they form networks that enable multicellular functions, altogether leading to differentiation and community-like living, termed biofilms. Bacterial biofilms confer advantageous survival mechanisms to community members, which often translate into virulence, pathogenesis, or resistance to antibiotics agents when looked at from the perspective of the host.^1^ The study of culturable skin-associated microorganisms, including species such as *Staphylococcus aureus* (*S. aureus*), *Staphylococcus epidermidis* (*S. epidermidis*), *Propionibacterium acnes* (*P. acnes*), *Malassezia spp.*, and others, has spanned decades and elucidated complex molecular mechanisms important to their skin associations.^2,3,4,5^ Classical microbiological and dermatological studies have reproducibly pointed to the strong associations between *P. acnes* and acne vulgaris, *S. aureus* and atopic dermatitis (AD), and *Malassezia* species with dandruff. In addition, many skin-associated microbes have been studied with regard to their biofilm-forming capabilities and efforts to hamper such biofilm production abounds.^6^ Despite that, direct linkage between skin microbes, their biofilm states, and disease has been scarce.

In this review, we focus on the connection between the skin microbiome, skin diseases, and biofilms of classical skin pathogens. We first describe several skin diseases and their microbial component from a classical microbiological perspective, and then move to summarize advances in skin microbiology as a result of the advent of next-generation sequencing technologies, with a specific focus on common skin diseases. Finally, we discuss future directions for studies of microbiological skin disorders, based on cutting-edge molecular biology techniques.

## Biofilms

The transition from planktonic to biofilm state begins with the attachment of microbes to a surface, which can be either living or abiotic. These immobilized communities range in size from small aggregates of tens of cells, to large biofilms encompassing hundreds of thousands of bacteria.^7^ The bacteria subsequently produce and excrete a variety of compounds to strengthen the attachment and expansion capabilities of the nascent community, collectively termed the extracellular matrix.^8^ Extracellular components of the bacterial biofilm consist of various biopolymers, including polysaccharides, DNA, proteins, and lipids.^9^ Recently, mineral scaffolds have also been shown to play a role in the assembly of the extracellular matrix.^10^ The biofilm state offers numerous advantages on the microbial community, principally by conferring a protected method of growth in an often hostile environment, whereby the biofilm community becomes less sensitive to antibiotics.^1^ The mechanisms of this biofilm phenomenon can be due to either the reduced diffusion rate of antibiotics through the extracellular polymeric substances, or through the reduced metabolic and altered phenotypic characteristics of the bacteria in the biofilm.^11^

Biofilms are often found in nature as multispecies or polymicrobial biofilms, coexisting within the larger framework of a broader community.^12^ As with single-species biofilms, polymicrobial biofilms formation is influenced by a number of factors, including the physiochemical surface environment, host receptors, nutrient availability, aggregation pattern, and local immune system activity.^7^ Finally, co-occurrence of different species within a habitat can involve various modes of interspecies communication, including quorum sensing.^13^

In vitro single-species biofilms of skin microbiota, such as *S. aureus*, *S. epidermidis*, and *P. acnes*, have been investigated at depth,^14^ yet *Malassezia spp.* biofilms remain relatively unstudied. Moreover, critical interspecies interactions have been uncovered with regard to the skin prokaryotes, including the inhibition of both *P. acnes* growth and *S. aureus* biofilms by *S. epidermidis*;^15,16^ however, these interspecies interactions have yet to relate to fungal commensals of the skin, principally *Malassezia*.

## Diseases, microbes, and biofilms

### *S. aureus* and AD

**Fig. 1:**
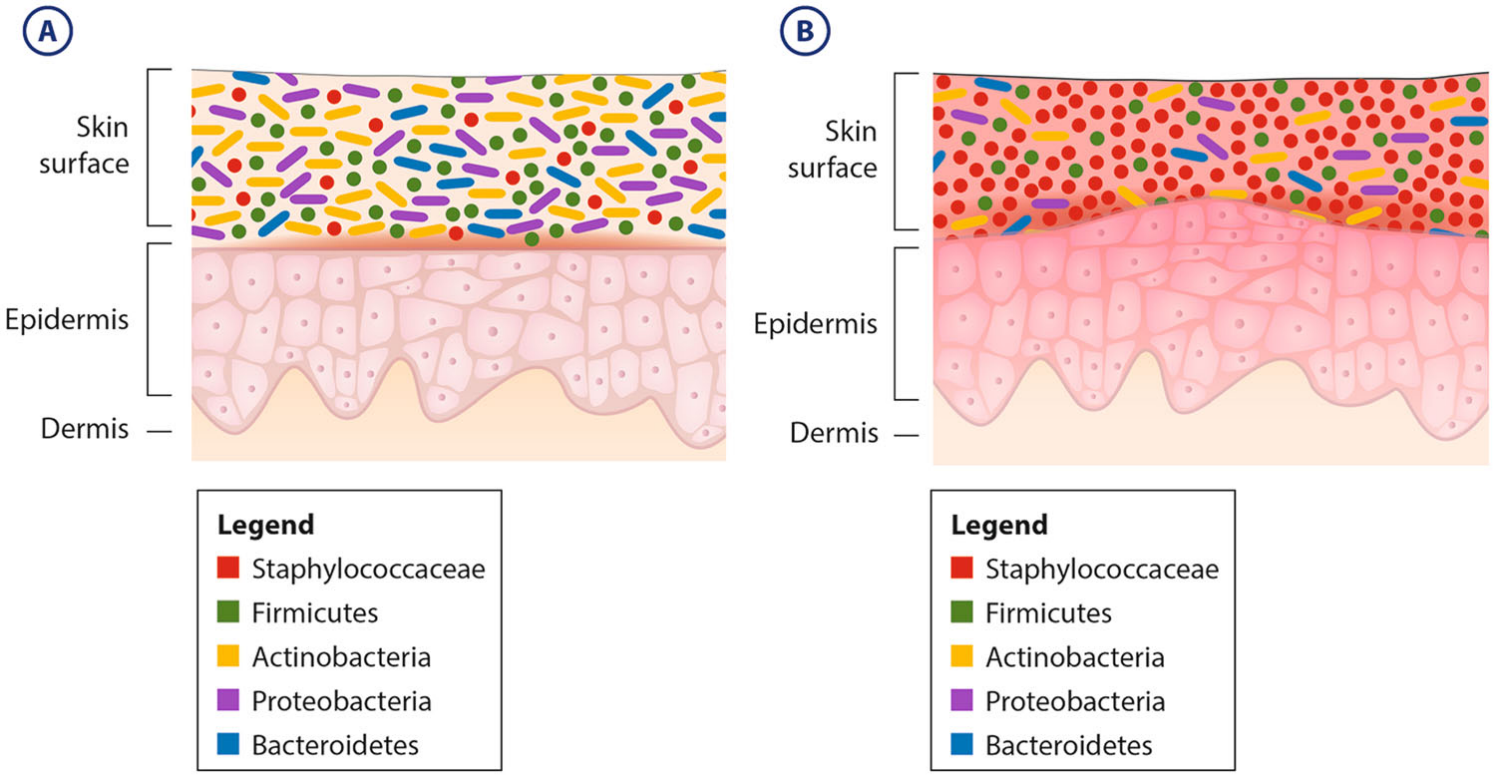
AD flares are characterized by shifts in relative abundances of several bacterial species. **a** Abundance of the dominant skin bacterial phyla (*Firmicutes*, *Actinobacteria*, *Proteobacteria*, and *Bacteroidetes*) and the family *Staphylococcaceae* (a *Firmicute*) associated with healthy skin. **b** AD induces dysbiosis characterized by a decrease in bacterial diversity and the dramatic increase in the proportion of *Staphylococcaceae* ^23,51^

AD, or atopic eczema, affects 20 % of children in westernized countries. The disease involves the recurrent appearance of inflamed, dry, and eczematic lesions on the skin and significantly impacts the quality of life of those affected.^17^ Two mechanisms have been proposed to explain the pathogenesis of the disease; one posits an immune defect, which leads to symptoms, while the other claims that the root issue lies in an inherent lacking in barrier function by epithelial cells, which leads to the immune response typical of AD lesions.^18^ The microbial etiology of *S. aureus* in AD has been the focus of much research since the original association between the two in 1974.^19^ Back then, *S. aureus* was found to colonize the skin of 90 % of patients with AD.^19^ The disease–microbe association has consistently been documented since, irrespective of environmental and therapeutic pressures such as antibiotics and geographic location.^20^ Skin colonization by *S. aureus* has been proven to exacerbate disease state through several immune-mediated mechanisms, thereby leading to inflammation and sensitization.^18,21,22^ A recent study had demonstrated an increased *S. aureus* abundance preceding flares in AD patients^23^ (Fig. 1). Additionally, topical and/or systemic antibiotics have been shown to facilitate the healing of flares, further concretizing the value of this host–microbe relationship in AD.^22^

*S. aureus* skin carriage and AD, while mostly not studied from the perspective of a biofilm, seems to fit snugly into the aforementioned survival parameters. *S. aureus* strains isolated from hospital settings and from patients in multiple body sites including the skin were demonstrated as biofilm producers.^24^ Moreover, the vast majority of *S. aureus* isolated from AD lesions were shown to be strong biofilm producers in vitro, and Congo red staining confirmed the existence of biofilms on skin.^25,26^ In a separate study, *S. aureus* biofilms were visualized on the stratum corneum of AD patients using scanning electron microscope (SEM), and the anti-biofilm compound farnesol was shown to decrease *S. aureus* carriage on AD patients.^27^ These results indicate a biofilm orientation of the *S. aureus* environmental factor in triggering and in the progression of AD. Interestingly, roughly 70 years ago, a group of dermatologists posited that AD begins with the occlusion of eccrine ducts by slime,^28^ which can undoubtedly be the extracellular polysaccharides known to exist in microbial biofilms. The presence of biofilm producing strains of *S. aureus* on the skin and in ducts may prove as a determinant of disease severity.

### *P. acnes* and acne vulgaris

The relationship between *P. acnes* and the disease acne vulgaris has been slow to develop and hard to pinpoint. Culiffe and Schuster first reported in 1969 the strong association between “greasy skin”, or increased sebum excretion on the skin surface, and acne vulgaris.^29^ Several years later, after reporting of significantly higher counts of *P. acnes* in adolescents with the disease compared to those without it, Leyden et al. hypothesized a connection between increased sebum levels and *P. acnes* colonization. Moreover, the group hypothesized a pathogenic role for *P. acnes* in the disease, yet emphasized that its etiology was no more than circumstantial given their findings.^30,31^ Antibiotic therapy often improves clinical presentation, and its failure to do so universally can be attributed to resistant strains of *P. acnes*.^32,33^ However, acne vulgaris can develop in the absence of *P. acnes* skin colonization, and another Propionibacterium strain, *P. granulosum*, has also been associated with the disease.^34^ To date, no clear mechanism has been elucidated to describe the microbiological etiology of acne vulgaris.

**Fig. 2:**
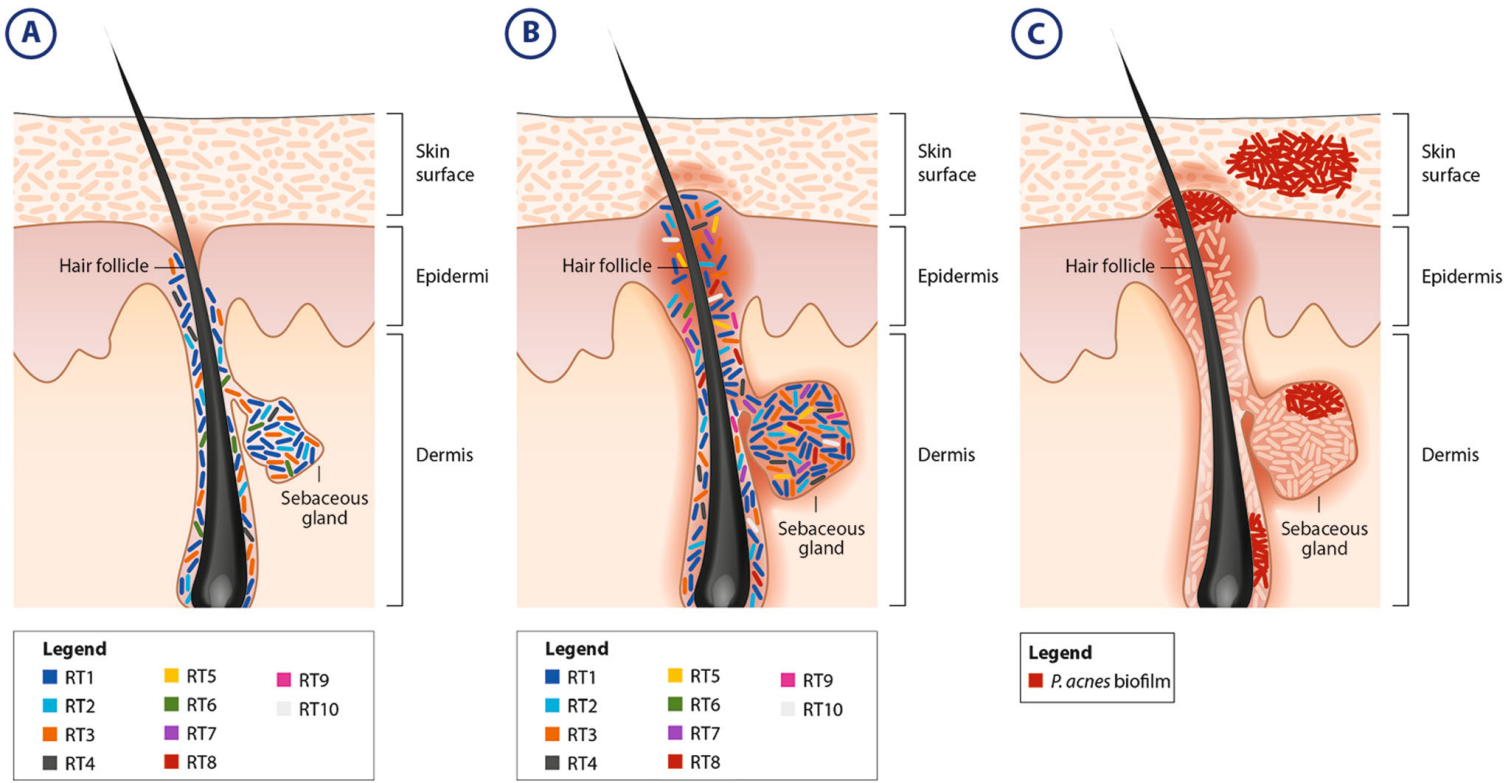
Relative *P. acnes* strain abundance in the nose pilosebaceous unit is different between acne patients and healthy individuals. **a** Normal relative abundance of dominant *P. acnes* strains in healthy individuals. **b** Acne-induced dysbiosis is characterized by a decrease in the relative abundance of *P. acnes* strains RT3 and RT6, and an increase in the relative abundance of strains RT4, RT5, RT7, RT8, RT9, and RT10.^53^ **c** The persistent nature of acne vulgaris and its ability to be only partially ameliorated through antibiotics can be due to pockets of biofilm-forming *P. acnes* strains located on various skin appendages, including on the skin surface, the sebaceous gland, the hair follicle, and the pore itself

Classically, the study of the association between *P. acnes* and acne has been limited to culture-based studies. Such studies have demonstrated the biofilm-forming capacity of *P. acnes* in vivo, yet the clinical relevance for such observations was hard to ascertain.^35^ Only recently researchers have developed new tools to uncover the relationship between this microorganism and the human host in vivo. Crucially, the introduction of tools to visualize *P. acnes* colonization of the skin and associated microenvironments has pointed to matrix-embedded communities at different levels of the skin. Fluorescent in situ hybridization and immunofluorescent microscopy have enabled us to picture macrocolonies and biofilms formed on the stratum corneum, sebaceous gland, and hair follicle walls of acne lesions.^36,37,38^ In fact, several groups have posited that the persistent nature of acne vulgaris is due to *P. acnes’s* colonization of the sebaceous unit in a biofilm, thereby eluding eradication by antibiotics (Fig. 2c). Such a theory posits treatment of acne via agents that will fundamentally alter the biofilm and/or its microenvironment.^39^ Support for this theory was granted with the report that the *P. acnes* genome contains genes that are responsible for the production of quorum-sensing molecules as well as extracellular polysaccharides (EPS).^40^ Additionally, isolates from invasive infections were stronger biofilm producers in vitro than isolates from healthy skin.^41^ Burkhart and Burkhart have further postulated that compounds produced by the *P. acnes* biofilms combine with sebum to cause keratinocytes adherence and eventually lead to clogging of skin pores.^42^ The incidence of biofilm-forming strains of *P. acnes* in selected microenvironments can potentially explain the incidence of the disease in a part of the population, despite the universal carriage of the microorganism. Moreover, it can account for the decrease in efficacy of antibiotics treatment observed in some acne patients and can be used as basis for the development of novel therapeutic agents that abrogate biofilm formation.^43,44^

## Next-generation sequencing and the skin microbiome in health and disease

The advent of next-generation sequencing technologies, and its adaptations to research skin microbial communities, has led to a dramatic shift toward a more complex and thorough understanding of the composition and extent of the natural human skin microbiota, as well as its functional capacity. While classical skin microbiological research had been dominated by the culturable organisms discussed above and their disease-related niches, we are now aware of nuanced skin niches and their colonization by specific taxa. Sebaceous, moist, and dry areas, typically independent dermatological environments associated with distinct disorders, are known to select for divergent communities. *Propionibacterium spp.*, long recognized as lipophilic colonizers of the sebaceous follicle, were shown to share their habitat with *Staphylococci*, *Corynebacteria*, and *β-Proteobacteria*, among others. The latter three were also shown to dominate moist sites, yet the lack of available triglycerides selects against colonization by *Propionibacterium*. Finally, dry skin harbors the most diverse phylotype, including varying relative abundances of *Firmicutes*, *Proteobacteria*, *Bacteriodetes*, and *Actinobacteria*.^45,46^ The aforementioned site specificity holds true for children and adults, yet the dominant taxa in adults vary from that of adolescents.^47^ Additionally, community membership has been shown to be similar throughout the epidermal and dermal layers, exhibiting greater beta-diversity than alpha-diversity.^48,49^ These significant observations have shattered the historical paradigm of the skin serving as an almost impermeable barrier for bacterial invasion, with select few bacterial species capable of exploiting its surface characteristics for colonization.^50^

Along with the new revelations of the composition of the human skin microbiome in health, several important studies have focused on the resident microbiota of certain skin diseases. The understandings of both AD and acne vulgaris were again brought into the spotlight, with new revelations and controversies arising. Kong et al., in their seminal work on the skin microbiome of AD, supported the conventional *S. aureus* association with the diseased state, and showed its correlation with disease severity. In addition, they showed that microbial diversity was reduced in the lesions, and that therapy and healing both increased diversity. Therefore, *S. aureus* colonization and overall microbial diversity were shown to be anti-correlated^23^ (see Fig. 1). Similar observations were made by observing the microbiome of affected and unaffected skin of patients with AD before and after emollient treatment.^51^ Using a mouse model, Kobayashi et al. proved that impaired epidermal growth factor receptor (EGFR) signaling can lead to dysbiosis, which in itself leads to eczematous inflammation, similar to that of human AD.^52^ Such an understanding of the role of the skin microbiome in AD supports initiatives to realize the power in harnessing, stabilizing, and directing the resident skin microbiota to establish a healthy state.

While molecular studies based on 16S rRNA sequencing have been successful in further expounding the microbiological etiology of AD, their application to study acne vulgaris has served to perpetuate the *P. acnes*–acne conundrum. For example, Fitz-Gibbon et al. reported no correlation between the relative abundance of *P. acnes* and acne, yet strain-level analysis revealed a strong connection between specific strains and the disease^53^ (Figs. 2a,b). In the 2 years since publishing their research, no group has elucidated the microbiome of other clinically relevant sites.

## Perspective

Although skin metagenomics studies have yet to bring therapeutics to market, the potential and utility of such studies have been firmly established. The feasibility of microbiome-based skin diagnosis has been proven for psoriasis patients.^54^ Many therapeutic approaches may be honed with our evolving knowledge of the skin microbiome. Currently, prebiotics, probiotics, and bacteriophages are being examined for their ability to steer microbial communities away from a state of dysbiosis and direct it toward a desirable healthy state.^55^ Additionally, the recent introduction of techniques to study the skin metabolome may strengthen the foundation for microbial-based forensics, diagnostics, and therapeutics.^56^ All of the aforementioned techniques and approaches can benefit greatly from a biofilm-focused approach toward characterization of the microbial component of the issue at hand.

Limitations to the observations made by 16S rRNA analyses are steadily being overcome. For one, molecular techniques based on the amplification of DNA do not differentiate between live, dead, and dormant community members, allowing only for a low-resolution snapshot of microbial life living on surfaces. This caveat in microbial community analysis was recently circumvented by Korem et al. who showed that by measuring read coverage and correlating it with the reads’ location in relation to the bacterial origin of replication, one can identify active and dormant members of a microbial community.^57^ This key advance in the ability to profile microbial communities can be translated to more meaningful analyses and deeper understanding of the microbial world. However, their approach fails to describe the reason for dormancy in a bacterial cell. Bacteria in the biofilm state can transition to a less active metabolic state, and therefore replicate at lower rates.^58^ In this state they may prove more resistant to antibiotics.^59^ Conversely, dead or dying cells will also not be in a replicating state. Therefore, the umbrella term “dormant community members” fails to differentiate between these two distinct physiological states.

Undoubtedly, technologies and techniques will surface in the coming years that will enable us to close the knowledge gap with regard to skin–microbe relationships. Such progress can be demonstrated clearly by the advancement made in characterizing the skin metagenome from the early days of the human microbiome project to this day. Lacking a systematic protocol for collecting sufficient amount of genomic material for whole genome shotgun (WGS) sequencing, the Human Microbiome Project members selected samples with at least 50 ng of DNA from among the pool of samples processed for 16S analysis, for WGS analysis.^60^ They reported WGS data from roughly 10 % of the samples collected, of which less than 20 % represented skin-associated samples.^61^ Most recently, Oh et al. successfully developed a pipeline for characterizing the human skin metagenome by individual topographical sites, capturing the milieu of microorganisms residing on the skin, including bacteria, viruses, and fungi.^62^ The characterization of the skin microbial transcriptome has eluded proper study due to the abundance of RNases found on the skin coupled with the relatively low bioburden of the skin microbiome. Similarly, it was not until this past year that the metabolome of the skin microbiota was elucidated.^56^ Transcriptomics and metabolomics of the resident skin microbiota may prove crucial in understanding key functions and activities of the skin microbiome and the changes they may exhibit under states of disease. Such characterizations will undoubtedly be the focus of many studies to come.

Critically, next-generation sequencing (NGS) technologies can be used to further understand the microbial biofilm nature of certain skin diseases. Global gene expression is altered in most bacteria when shifting from the planktonic to biofilm state.^63,64,65^ Therefore, specific genes, or entire transcriptomes, can be used to determine whether the microbial inhabitants of clinically relevant specimens appear as free-living or community-oriented organisms. RNA-seq, or whole transcriptome shotgun sequencing, has been adopted to study the gut,^66^ oral,^67^ and environmental transcriptomes,^68^ yet has remained unstudied in the context of the skin. This obscurity may have been a result of the lack of a database containing reference genome sequences for skin bacteria and the low bioburden of microbial species on skin.^46^ However, Oh et al.’s recent work on skin metagenomics have paved the way to overcome the former issue, while steadily decreasing input nucleic acid requirements of commercially available kits have helped to overcome the latter issue.^62^ Similarly, proteins and metabolites unique to the biofilm state can be used as biomarkers for disease types and severities.

Novel techniques based on our understanding of bacterial biofilms may lead to therapeutic strategies as well. It has been documented extensively that antibiotics can deplete certain elements of the gut microbiota^69^ and that quorum sensing plays an integral role in bacterial communal living.^70^ Thompson et al. recently balanced the two ecological factors by first selectively depleting *Firmicutes* from the gut-microbiota of antibiotics-treated mice using streptomycin and subsequently introducing an *Escherichia coli* strain engineered to overproduce auto-inducer 2 (AI-2), a universal yet species-specific quorum-sensing molecule, into the mouse gut. Consequently, the AI-2 favored the propagation of *Firmicutes* over *Bacteroidetes*, opening new and tantalizing quorum-sensing-assisted therapeutic tactics.^71,72^ Another promising therapeutic strategy that is gaining attraction in the scientific world is phage therapy.^73^ Recent studies have demonstrated the potential in using bacteriophages to combat biofilm-related diseases, thanks to their ability to penetrate the deeper layers of biofilms and to target antibiotics resistances that develop in the context of biofilms.^74,75^ Additionally, the recent elucidation of the human skin virome and its integration into our understanding of the human skin microbiome may result in new avenues for directed perturbations of biofilms and or microorganisms involved in disease or dysbiosis.^76^

## Conclusion

The skin is the largest organ in the human body and forms an immense interface between the host and its environment. As such, it constitutes an important site of interactions between the immune system and its microbial inhabitants. Recent studies have established the link between the development of the resident immune system of the skin and the skin microbiota and have demonstrated a direct contact between the two (reviewed in Belkaid and Segre^77^).

Several bacterial species isolated from human skin have demonstrated their ability to form biofilms both in vitro and in vivo. In their biofilm state, bacteria present differential metabolic and physiological functions often rendering them more virulent and resistant to antibiotics. In this state they may be involved in the etiology and exacerbation of skin disorders. Such studies, in combination with culture-independent sequencing techniques, are now beginning to uncover the complexity of the skin microbiome and its functions as they relate to common skin disorders. Consequently, skin disorders that were classically regarded as non-infectious may prove to include an infectious component, or to be affected by one or more microbial agents.

Classically, research on the involvement of bacterial biofilms in skin diseases had concentrated mainly on chronic wounds, where the presence of biofilms have been linked to wound development, infections, and impaired healing. Additionally, the role of quorum sensing, or bacterial cell–cell communication, a significant regulator of biofilm formation, has been expounded partially in relation to bacterial infections.^78^ Direct evidence linking the extent of microbial burden and the presence of biofilms to the severity and prognosis of chronic wounds have been reported, but was limited due to the reliance of studies on culture-based techniques, resulting in low representations of species diversity and partial reflections of the nature of the microbial colonization.^79^ Modern studies on chronic wounds have attempted to overcome these limitations by the use of 16S rRNA sequencing for bacterial community analysis.^80,81^ Despite the fact that such studies have been complicated and influenced by the different sampling methods, the large variability of wounds, and the patients’ clinical status,^79^ they have contributed to a better understanding of chronic wounds and have demonstrated the importance of microbial biofilms in wounds development, and their implications to wound care and recovery. In this review, we have focused on the role of biofilms in skin disorders that are considered non-infectious and are not manifested as open skin wounds. In this context we have used two common skin disorders as illustrations of the importance of studying the involvement of biofilms to uncover aspects of the pathogenesis of skin disorders. Evidence for such involvement, though still limited, highlights the potential in studying the relationship between the occurrence and the development of biofilms and the etiology and/or exacerbation of skin diseases. Some limited evidence for the involvement of biofilms has been reported for additional skin disorders such as bullous impetigo, furuncle, and pemphigus foliaceus.^26,82^ Such reports further demonstrate that microorganisms can develop biofilms that are able to grow and persist on skin surfaces and appendages, necessitating further investigations.

Skin-associated bacteria have evolved to grow on specific skin niches, and their mechanisms of attachment, survival, and propagation on skin are only partially understood.^83^ Possible future studies should include measuring the expression of biofilm-related genes in diseased lesions, and/or using proteomics methods to characterize microbial biofilms on the skin. Additionally, the effects of toxins, metabolites, and other compounds secreted by microbial biofilms should be investigated with respect to the responses they invoke in skin keratinocytes or immunocytes. Lastly, inducing biofilm growth on ex vivo or in vivo skin models, and subsequently profiling the physiological and molecular adaptations undertaken by both the organ and the microbe, can further our understanding of the skin–biofilm relationship and potentially lead to the development of novel therapeutics.
